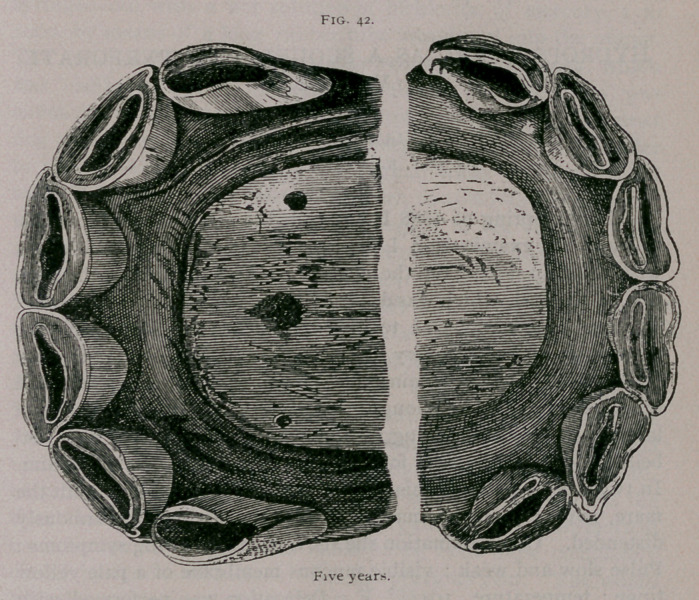# Age of the Horse, Ox, Dog, and Other Domesticated Animals

**Published:** 1890-09

**Authors:** R. S. Huidekoper

**Affiliations:** Veterinarian


### AGE OF THE HORSE, OX, DOG, AND OTHER DOMESTICATED ANIMALS.

By R. S. Huidekoper, M.D., Veterinarian.

*Continued from page 462.*

Third Period.—Eruption of the permanent or adult teeth.

This period commences at the age of two or two-and-a-half years and finishes at five years.

*About Two-and-a-half Years.*—Successive falling out of the temporary pincher teeth; swelling of the gums and appearance of the interior border of the permanent pinchers. Ordinarily these teeth appear in the superior jaw first, and the piercing of the gums is completely effected in about six weeks to two months.

*Rising Three Years.*—As the colt is approaching the age of three years, in the superior jaw we find the permanent pinchers wholly out of the gums and almost reaching the level of the temporary intermediate teeth. In the inferior jaw, the borders and sometimes a greater extent of the free extremity of the tooth has appeared through the gums, though the teeth are still virgin; the intermediate temporary teeth are free from the gums at their neck and very much worn. The corner teeth are worn so that they touch each other by their external borders (Fig. 36).

*Three Years.*—At three years of age all of the permanent pinchers are out of the gums and have reached the level of the temporary teeth. The permanent pinchers are wider transversely and darker in color than the temporary teeth, and show little gutters on their anterior face. They differ distinctly from the others, which are smaller, more convex, have a constriction in the neighborhood of the gums, and are wider and contain no gutters.

The time of the year and the race of the animal must be taken into consideration in determining the completion of three years. The better breed of horses attain that age in mid-Winter, while those of more common race do not attain it until the months of March, April or May.

*Three Years Off.*—When the colt is several months from three years of age, the permanent teeth are well used on their borders and in contact with each other, but the dental cup is not yet a complete circle, as the enamel which forms it is still connected with the peripheral enamel toward the border of the tooth. The intermediate teeth are very much worn, protrude from the gums and are sometimes broken. The table of the corner teeth has become very much larger and almost covers the external border of the tooth (Fig. 37).

*Rising Four Years.*—Eruption of the permanent intermediate teeth and progressive falling out of the temporary intermediate teeth mark this period. The permanent intermediate teeth appear, but are not yet worn and have not yet reached the level of the table of the corner teeth. The central enamel in the pincher teeth surrounds the dental cup, which is flattened from in front to behind, and is almost distinct. The corner teeth commence to be free at their neck from the gums (Fig. 38).

*Four Years.*—Each jaw shows four permanent teeth, with their tables on the same level; the intermediate teeth are worn both on their anterior and posterior borders, but the dental cup is not entirely separated from the outside peripheral enamel. Often the inferior pincher teeth are leveled, especially in well-bred horses. The temporary corner teeth stand out from the gums and are completely worn (Fig. 39).

*Four Years Off.*—Loosening and successive falling out of the temporary corner teeth, which are worn to stumps, scarcely fastened their alveolar cavities. Sometimes one or more of the corner teeth has fallen out, and we find the inferior border of the permanent corner teeth appearing first more frequently in the upper jaw. The pinchers and intermediate teeth are well worn. At this period we frequently find anomalies in the eruption of the teeth. It is not rare to see the intermediate and corner teeth appear at the same time, so that an animal which is only four or four-and-a-half years of age may have the teeth which ordinarily indicate five years (Fig. 40).

*Rising Five Years.*—The four temporary corner teeth have fallen out and are replaced by the adult teeth. These last have not yet reached the level of the intermediate teeth and are not yet worn. The pinchers are leveled; their central enamel, elongated from side to side, is found further and further away from the anterior border of the dental table. The table of the intermediate teeth is distinctly formed (Fig. 41).

*Five Years.*—The mouth is complete; the incisive arch is semicircular and regular in shape; all of the permanent teeth have reached the same level. The inferior border of the corner teeth is completely worn. The posterior is not yet worn (Fig. 42).

*Five Years Off.*—The above characteristics are more distinct. The age has appeared to be more marked by the continual friction and amount of work to which the corner teeth have been subjected. In the superior incisive arch, the posterior border of the corner teeth rarely commence to be worn. The profile of the incisives shows a regular one-half circle, covered in from above to below.

FIG. 36.

FIG. 36.

Rising three years.

From in front, in the upper jaw, the two permanent pinçher teeth are seen not yet opposite to the level of the intermediate deciduous teeth ; the adult pinchers are just coming through the gum, showing a small portion of their anterior face. In profile, the intermediate teeth are very much worn and the constriction on their neck is pushed out beyond their gums ; the corner teeth are much shortened ; the dental table shows slight wear of the superior pinchers, which has been produced by the eruption of these teeth before the inferior temporary pinchers had fallen out, and, consequently, they have worn against the latter. The intermediate teeth are completely leveled ; the corner teeth are much used.

FIG. 37.

FIG. 37.

Three years off.

From in front the four permanent pincher teeth are seen, much larger and stronger than the neighboring teeth. The anterior borders of the superior pinchers are. oblique, and their external borders are not yet in contact with the corresponding part of the inferior teeth. In profile, the intermediate teeth are seen much used ; the corner teeth are short, and show the contraction at their neck. The tables are worn off square. Between the corner and the intermediate teeth on the left is seen the protrusion of gum made by the permanent intermediate tooth which is shortly to appear. The dental tables of the inferior intermediate teeth are very much worn, the superior teeth somewhat less so. The inferior corner teeth are entirely leveled. In this mouth the tables of the inferior pincher teeth are the most worn, as these teeth came out before the superior.

FIG. 38.

FIG. 38.

Rising four years.

From in front are seen the four adult incisors. The pinchers in contact with the opposite ones, and the intermediate teeth not yet down to the level of the pinchers. In profile, this is also seen in this mouth. The corner teeth are very much worn; the tables of the pinchers are considerably worn, making almost a complete separation of the central or cup enamel from the peripheral enamel. This advanced wear of the pincher teeth is not in direct harmony with the amount of use of the corner teeth.

FIG. 39.

FIG. 39.

Four years.

From in front the four superior permanent teeth are seen in contact with the inferior teeth. The jaw has attained a width so that the corner teeth are almost hidden. In profile, the latter are seen to be very small. The superior ones have commenced to be pushed out from the jaw. In the lower jaw are seen the tush teeth. The tables of the intermediate teeth are much worn, especially in the upper jaw, in which the eruption took place first. The central enamel is only separated in the superior left-hand teeth; the inferior corner teeth are almost leveled, the superior completely so. The latter are being pushed out, and show their roots.

FIG. 40.

FIG. 40.

Four years off.

In front the intermediate permanent teeth are seen in contact with each other; the inferior and superior left-hand permanent corner teeth have appeared. In profile, it is seen that these teeth have not been completely pushed through the gums. The tush teeth have appeared. The right-hand superior milk teeth are ready to fall out; nothing remains but their roots. The inferior teeth on the same side are leveled, but still firmly imbedded in the jaw. The superior intermediate teeth, which had preceded the eruption of the inferior teeth, show considerable wear. The cups have formed in the pincher teeth.

FIG. 41.

FIG. 41.

Rising five years.

The four corner temporary teeth have been replaced by the permanent teeth, but these are not on the level with the intermediate and are entirely virgin. The tables of the other teeth show a more decided use than in the previous figures. Above, the cups are formed in both the pincher and intermediate teeth ; the cups are entirely formed in the inferior intermediate teeth.

FIG. 42.

FIG. 42.

Five years.

The mouth is entirely made; all of the permanent teeth have reached the same level in both jaws. The lower are convex in both directions; the tush teeth have completed their eruption. The corner teeth have commenced to wear on their anterior border; the pincher teeth are leveled, but the cup is still elongated from side to side and is very narrow; they are rather close to the posterior border of the teeth. This form of cup indicates that in these teeth the cup is very shallow. The incisive arches form a regular half circle.

(TO BE CONTINUED.)

## Figures and Tables

**Fig. 36. f1:**
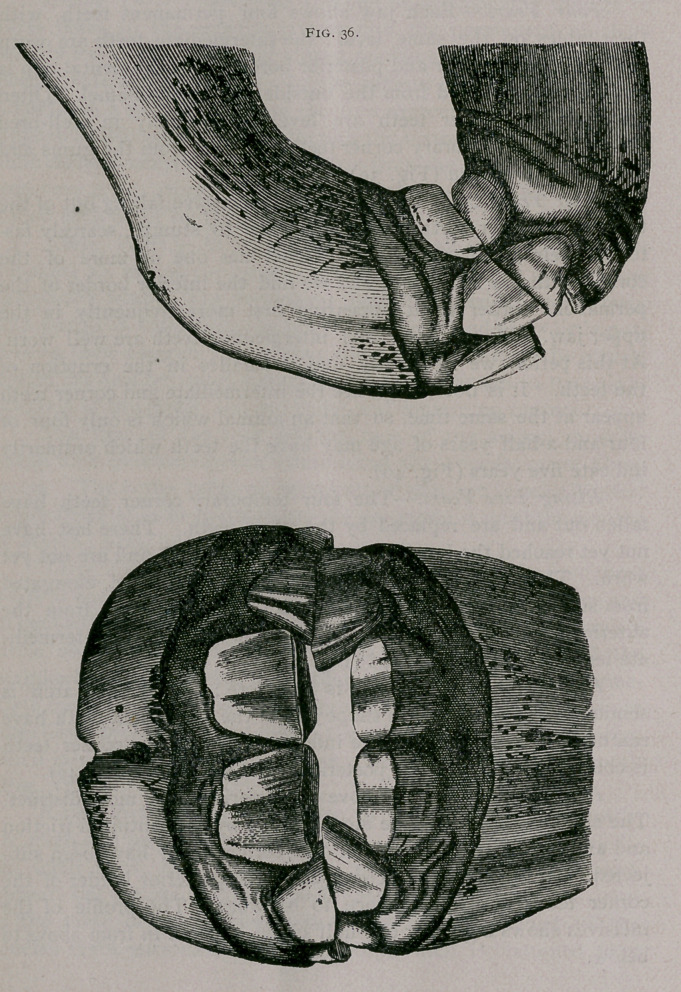


**Fig. 36. f2:**
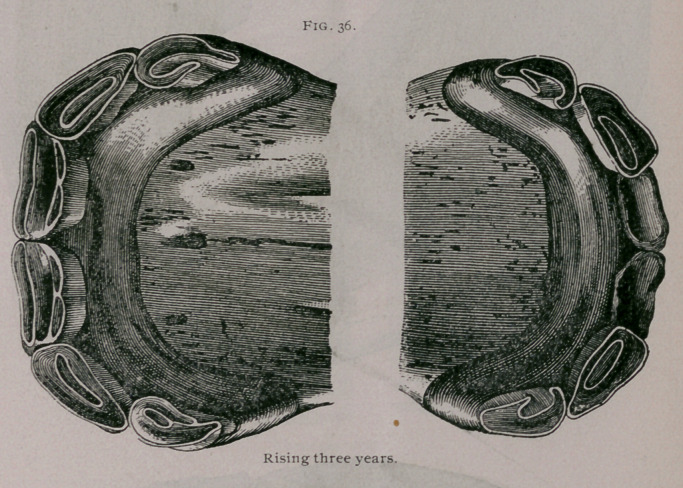


**Fig. 37. f3:**
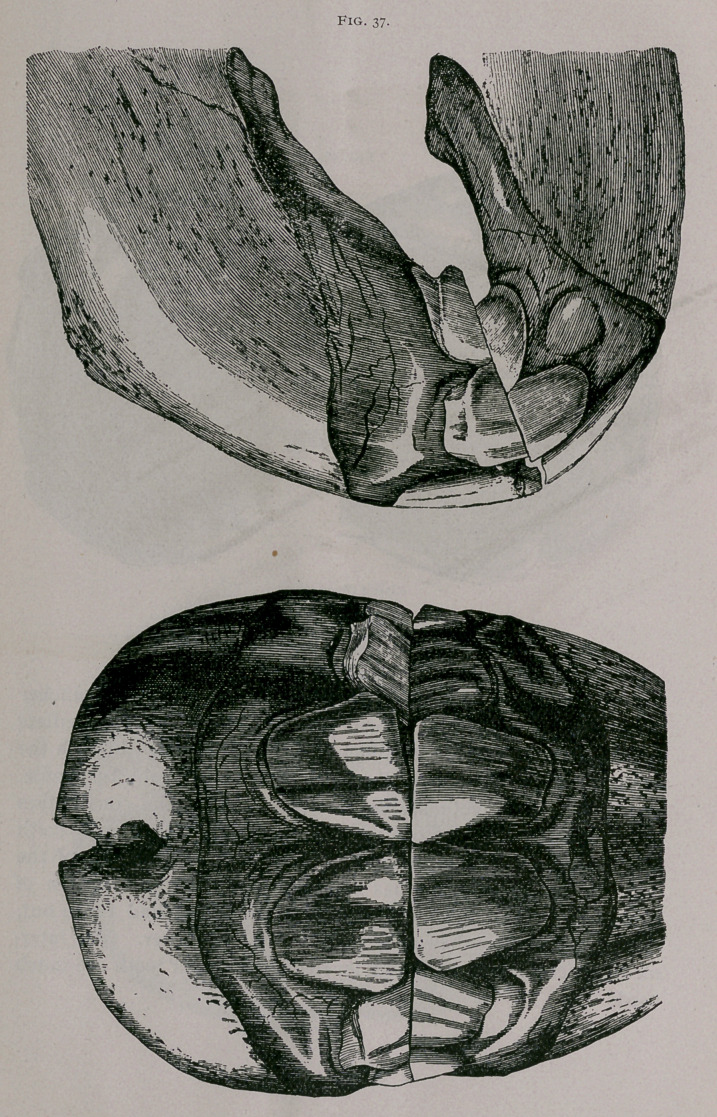


**Fig. 37. f4:**
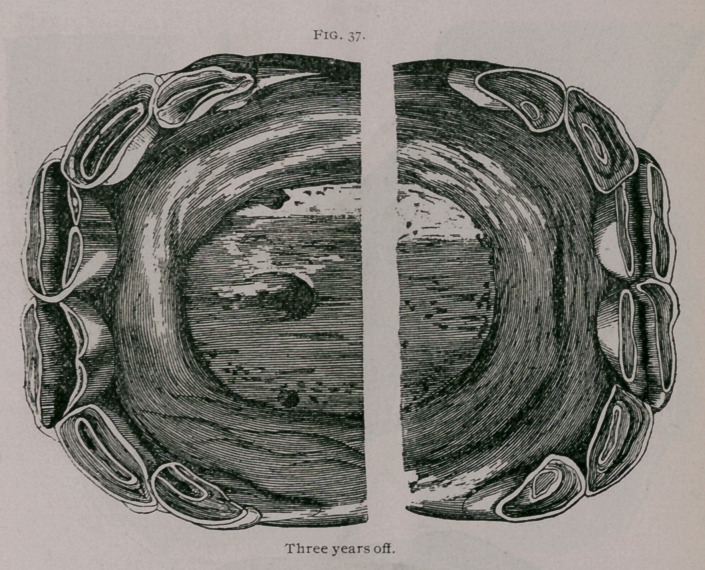


**Fig. 38. f5:**
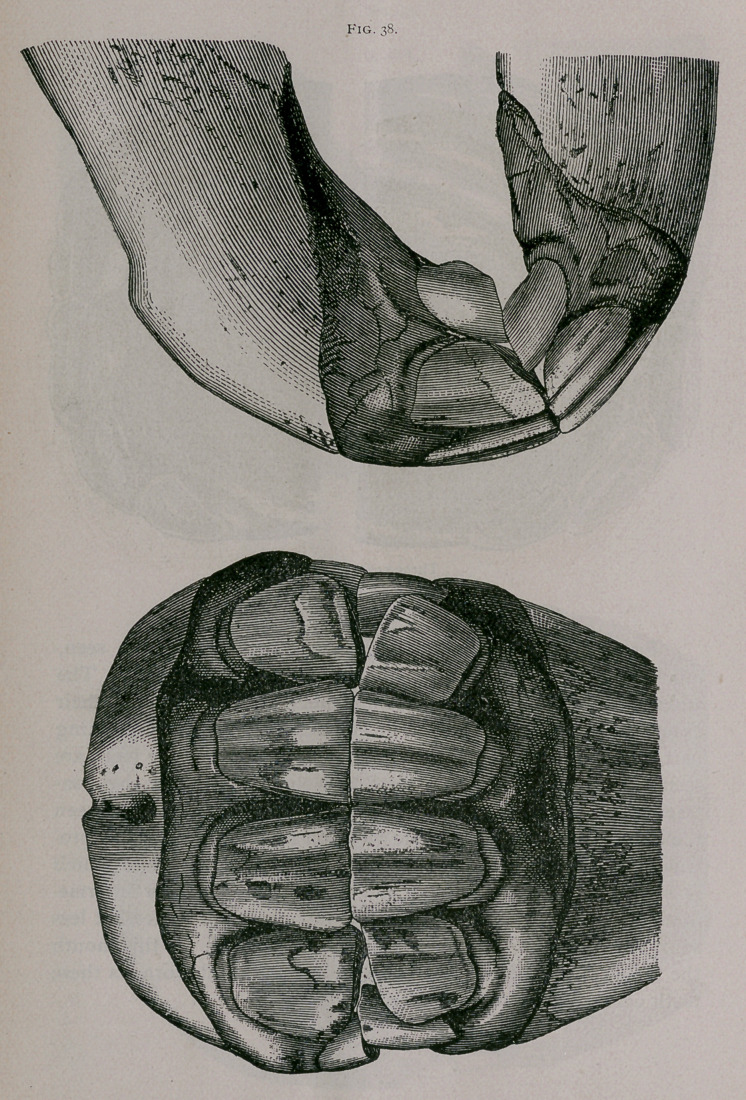


**Fig. 38. f6:**
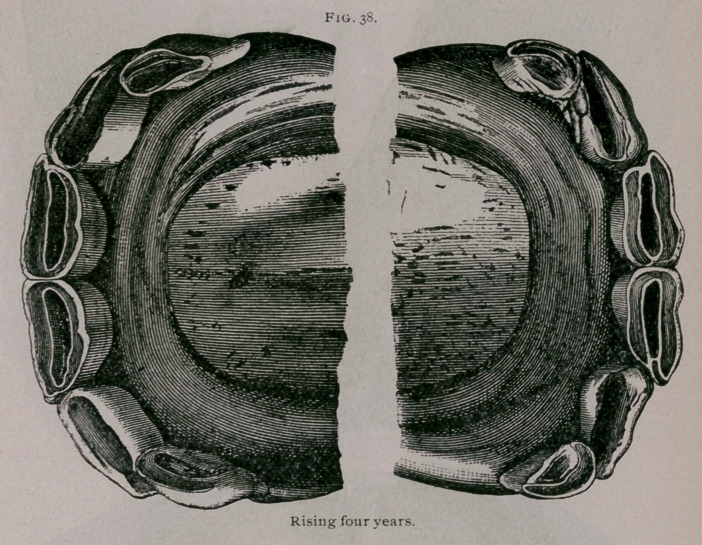


**Fig. 39. f7:**
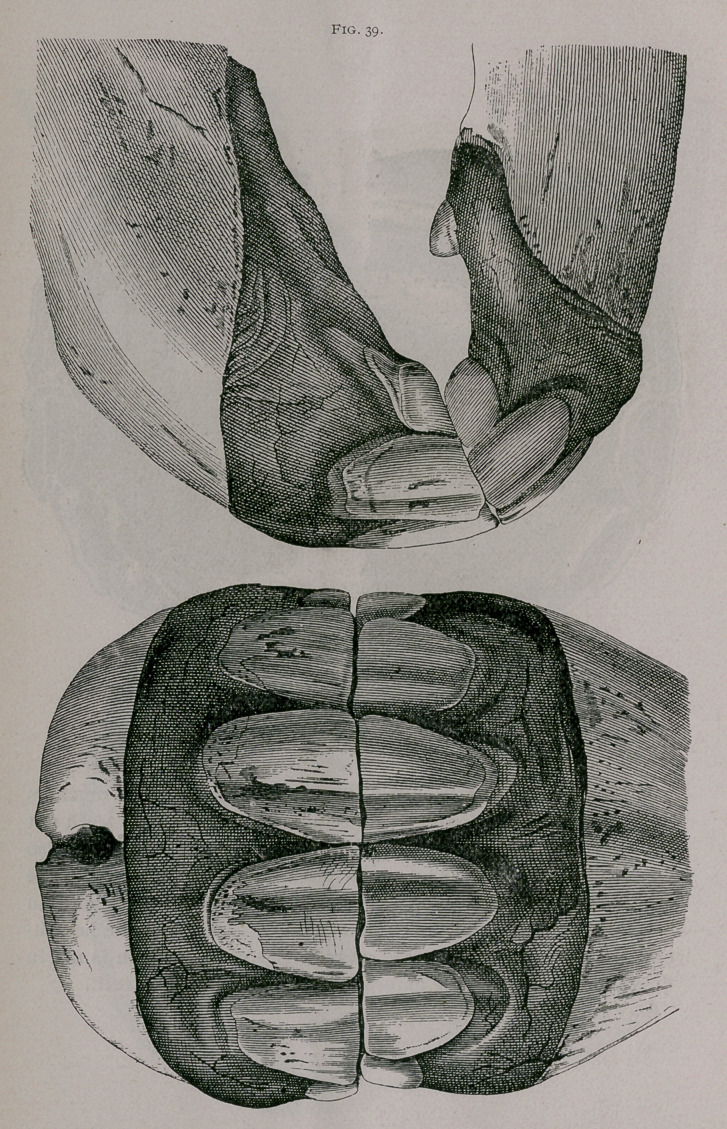


**Fig. 39. f8:**
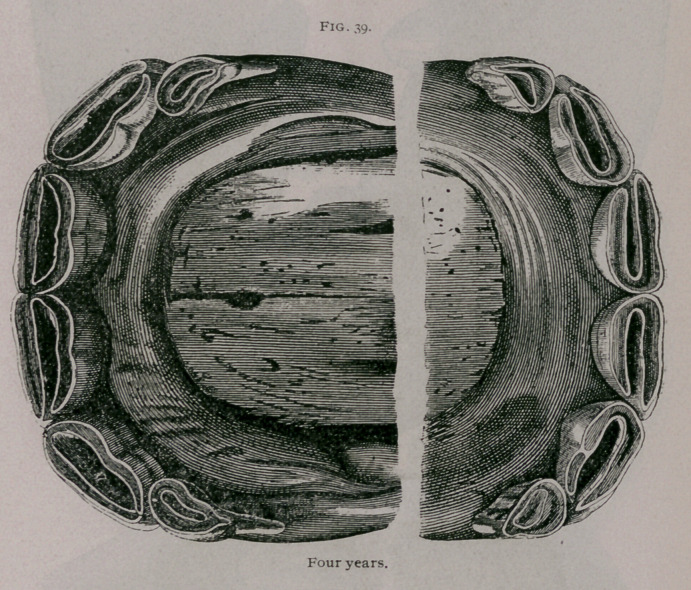


**Fig. 40. f9:**
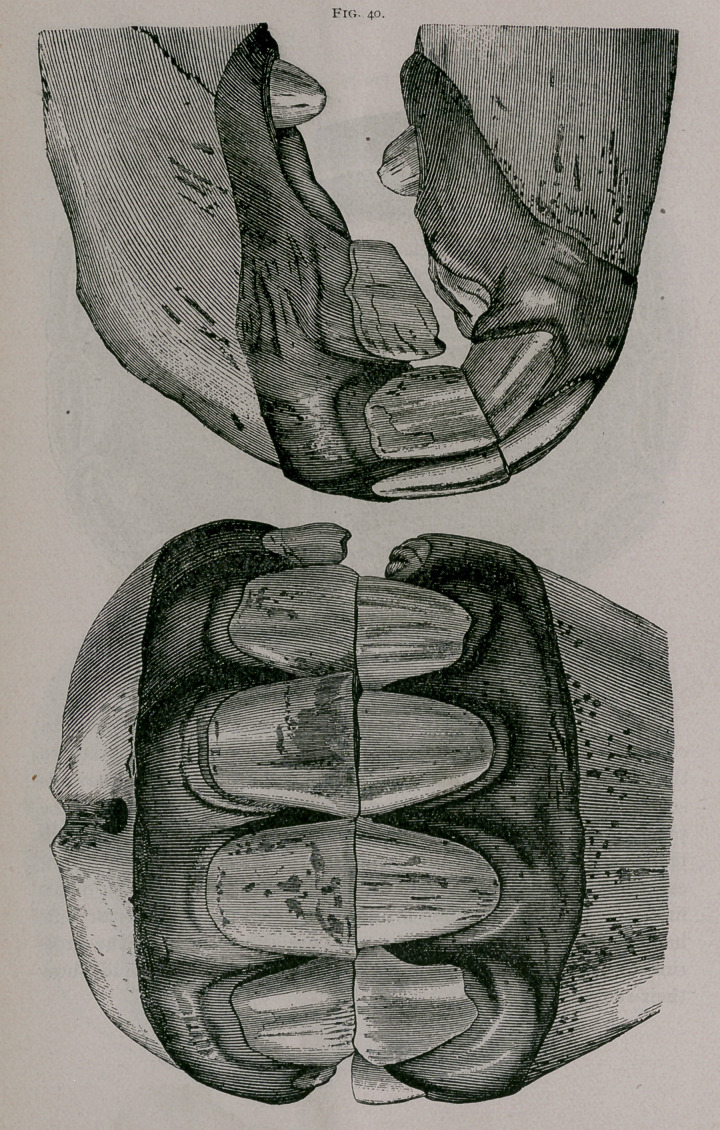


**Fig. 40. f10:**
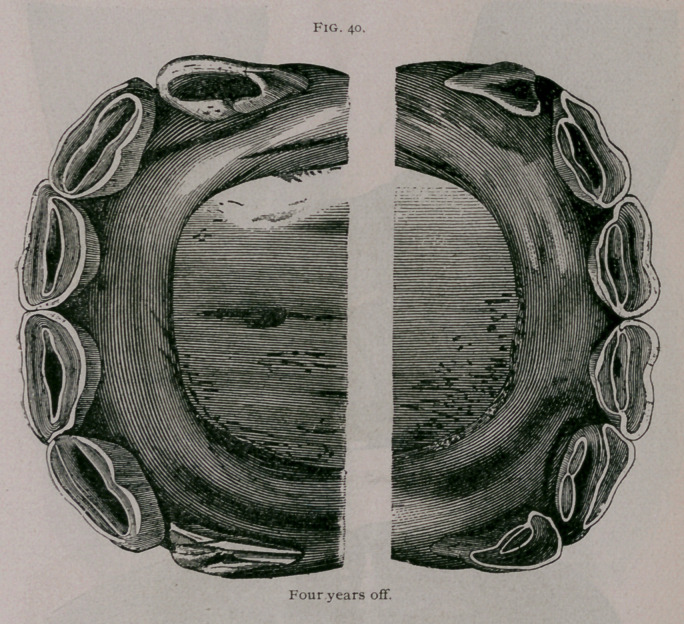


**Fig. 41. f11:**
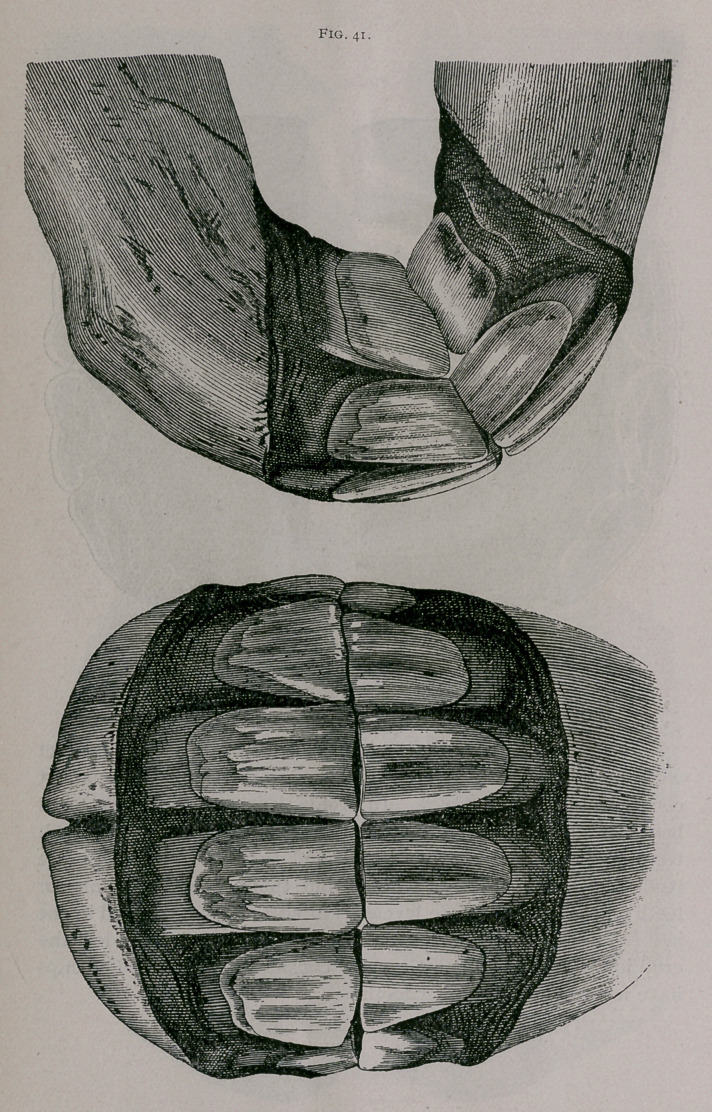


**Fig. 41. f12:**
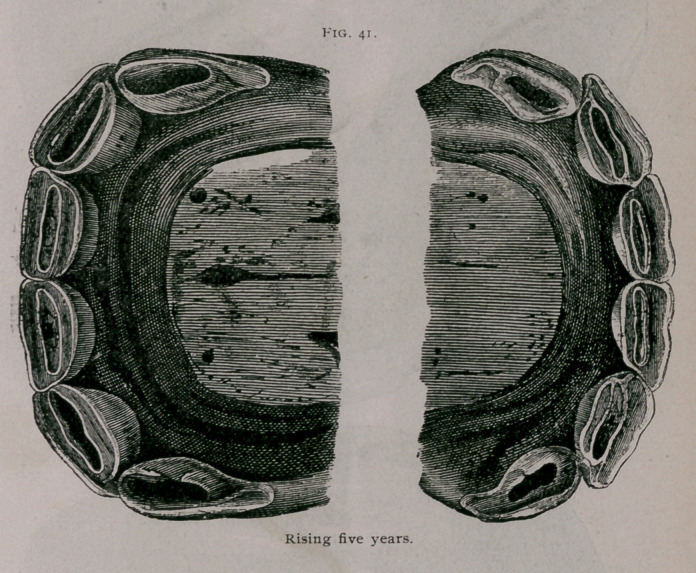


**Fig. 42. f13:**
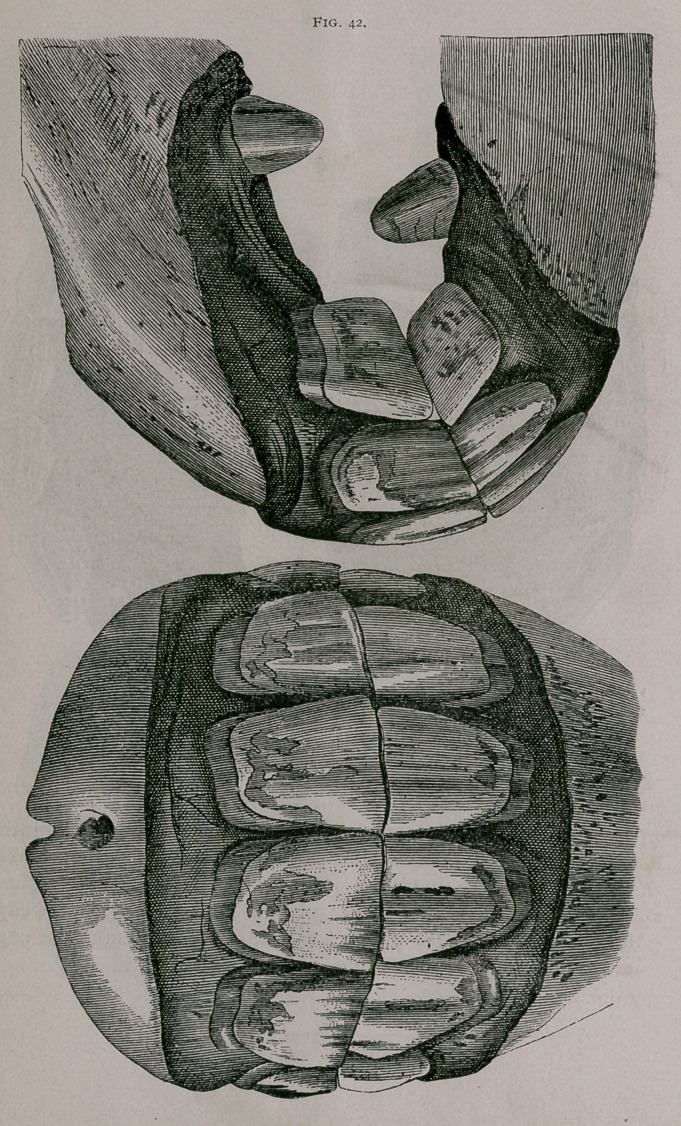


**Fig. 42. f14:**